# WHEN DO PAID CAREGIVERS SUPPORT THE HEALTH OF OLDER ADULTS? GERIATRICIAN PERSPECTIVES

**DOI:** 10.1093/geroni/igae098.2179

**Published:** 2024-12-31

**Authors:** Jennifer Reckrey, Leah Estrada, Deborah Watman, Emily Franzosa

**Affiliations:** Icahn School of Medicine at Mount Sinai, Icahn School of Medicine at Mount Sinai, New York, United States; Icahn School of Medicine at Mount Sinai, New York, New York, United States; Icahn School of Medicine at Mount Sinai, New York, New York, United States; James J Peters VA Medical Center, Bronx, New York, United States

## Abstract

Paid caregivers (e.g., home health aides, personal care attendants, other home care workers) may support not only the function but also the health of the older adults they care for in the home, but little is known about how medical providers view paid caregiver role in healthcare. This secondary qualitative analysis of interview data from geriatricians (n=9 geriatricians, n=27 interviews) used thematic analysis to identify geriatrician perspectives on when paid caregivers do the most to support the health of their older adult clients. Geriatricians perceived that paid caregiver contributions were greatest in the care of high-needs older adults including those with multimorbidity, dementia, and significant functional impairment. They described that paid caregivers stepped up to fill healthcare gaps when families could not provide all needed support (e.g., long distance family caregivers). They also described how the personal qualities and internal motivation of paid caregivers affected their role in care, but rarely acknowledged the systemic barriers to greater paid caregiver participation in health-related care. As the locus of long-term care continues to shift from institutions to the community, a greater number of older adults with multimorbidity and dementia will rely on paid caregiver to live at home despite growing functional impairment. Future work should consider how to best integrate paid caregivers who are already providing health-related support into the care team and explore barriers to paid caregiver participation in health-related care more generally, especially for older adults with the highest care needs.

